# Supplementary figures and images for: Wheat Straw Return Influences Soybean Root-Associated Bacterial and Fungal Microbiota in a Wheat–Soybean Rotation System
Source: Microorganisms. 2022 Mar 21;10(3):667. doi: 10.3390/microorganisms10030667 (PMC8951542; doi:10.3390/microorganisms10030667)

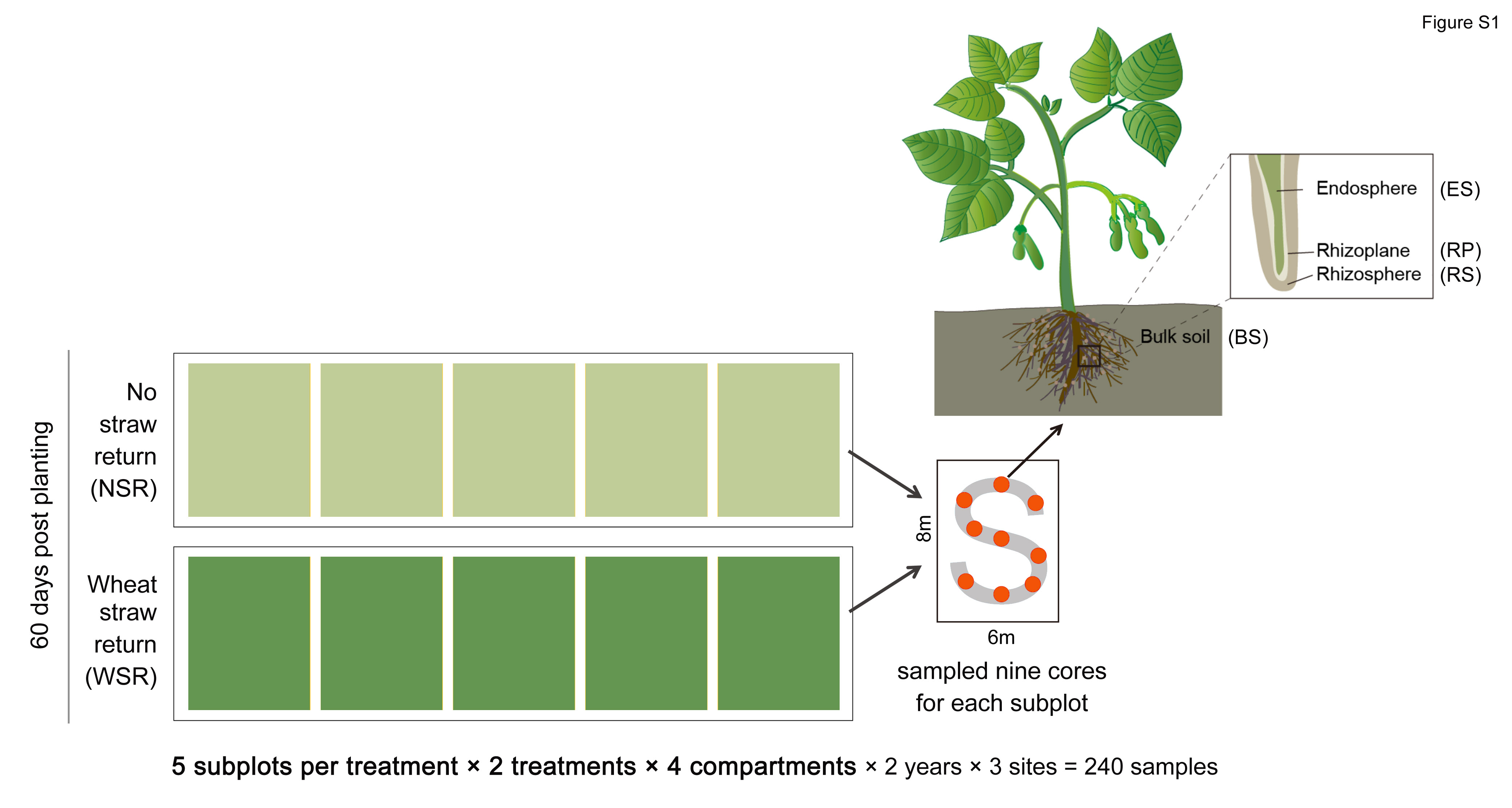

Supplement: Supplementary file 1 [file microorganisms-10-00667-s001.zip › Supplementary File/Fig S1.JPEG]

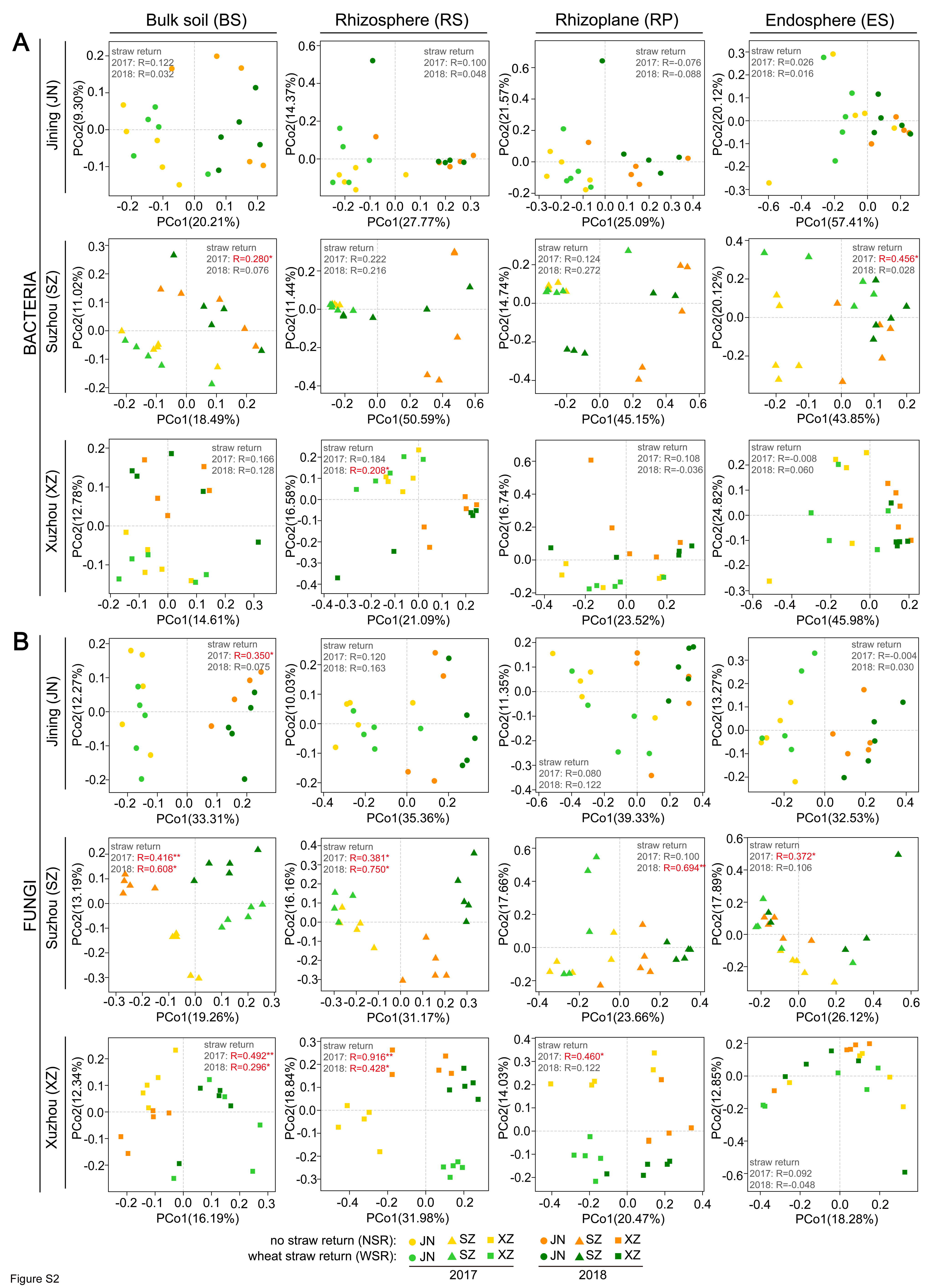

Supplement: Supplementary file 1 [file microorganisms-10-00667-s001.zip › Supplementary File/Fig S2.JPEG]

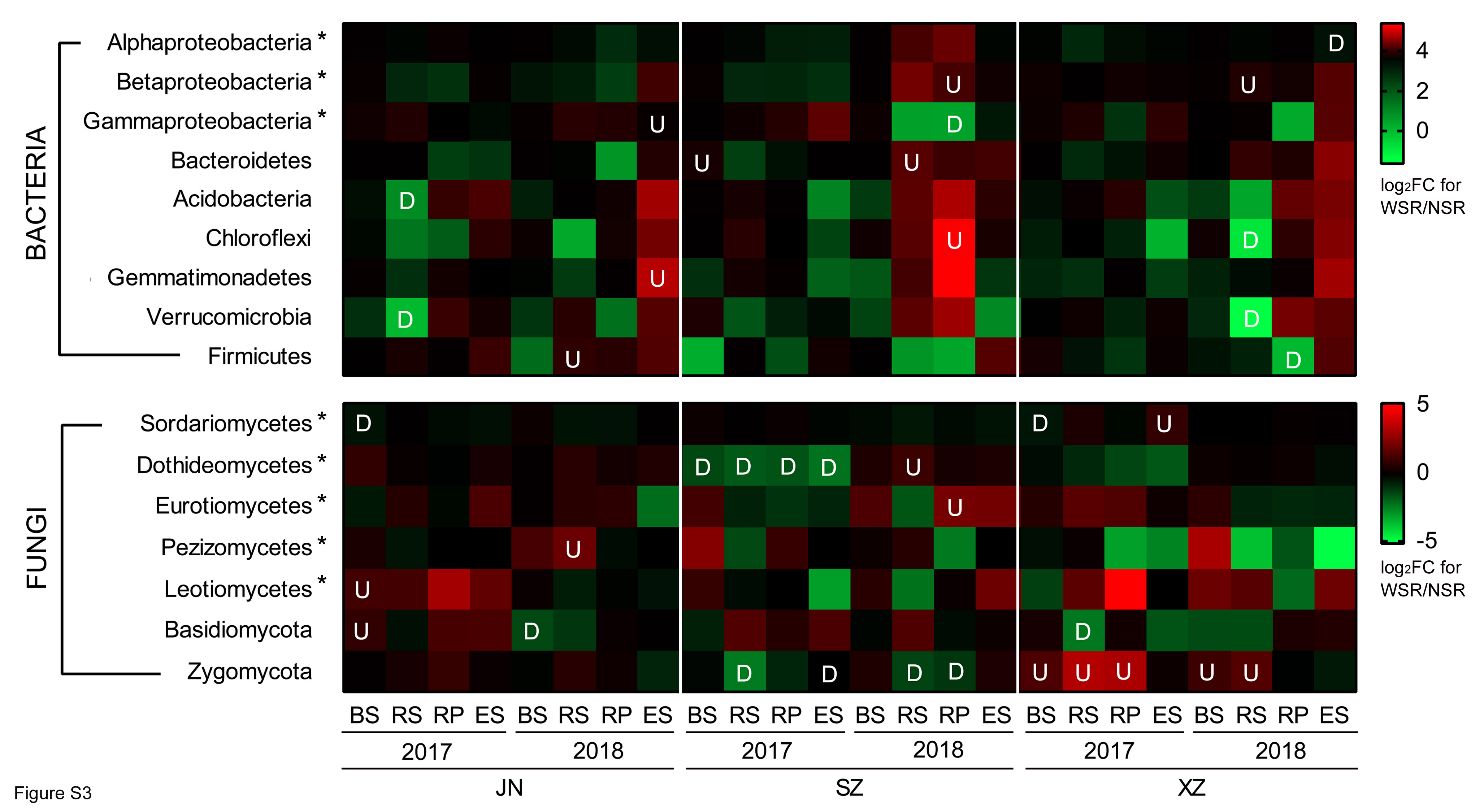

Supplement: Supplementary file 1 [file microorganisms-10-00667-s001.zip › Supplementary File/Fig S3.JPEG]
